# Supplementary material for: Differential Effects of Selenium Compounds on Mitochondrial Function in PRRSV-Infected Porcine Alveolar Macrophages
Source: Viruses. 2025 Sep 26;17(10):1303. doi: 10.3390/v17101303 (PMC12568000; doi:10.3390/v17101303)
Supplement: Supplementary file 1 [file viruses-17-01303-s001.zip › Final Supplementary Figures.pdf]

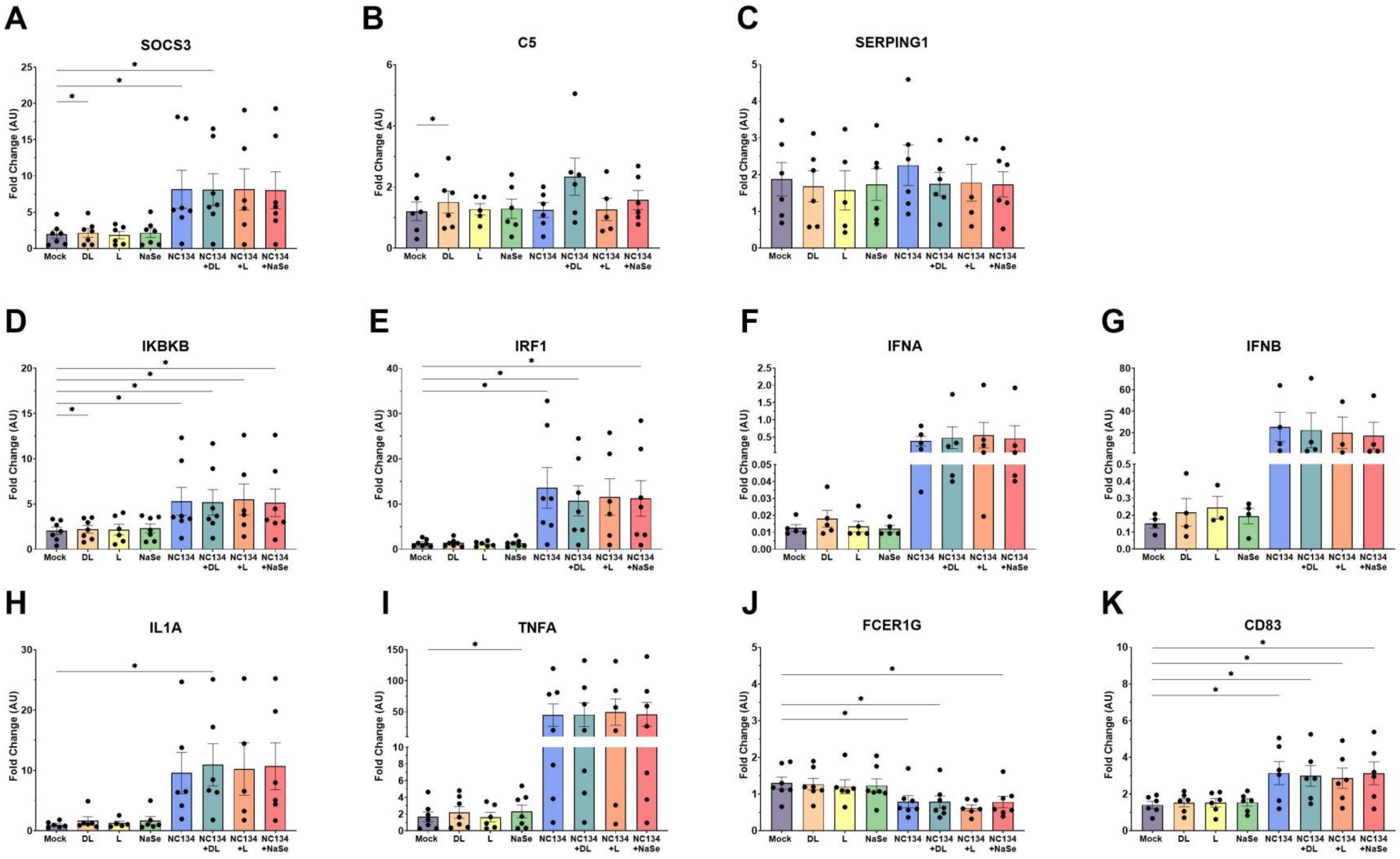

Figure S1. Immune cell gene expression analysis of PRRSV-2 NC134 and Se-compound treated macrophages, via NanoString technology. Cells were infected with NC134, treated with organic or inorganic Se, or left in the media alone (mock). Obtained values were normalized to the mock and shown as Fold Change (AU). Fold changes of (A) SOCS3, (B) C5, (C) SERPING1, (D) IKBKB, (E) IRF1 (F) IFNA, (G) IFNB, (H) IL1A, (I) TNFA, (J) FCER1G, and (K) CD83. All experiments were performed using between 3-7 biological replicates per experiment. Each symbol represents one biological replicate. Statistical significance was determined by comparing the means of all independent groups using One Way ANOVA. Results displayed showing mean and standard error of the mean (SEM). *p≤0.05, **p≤0.01.

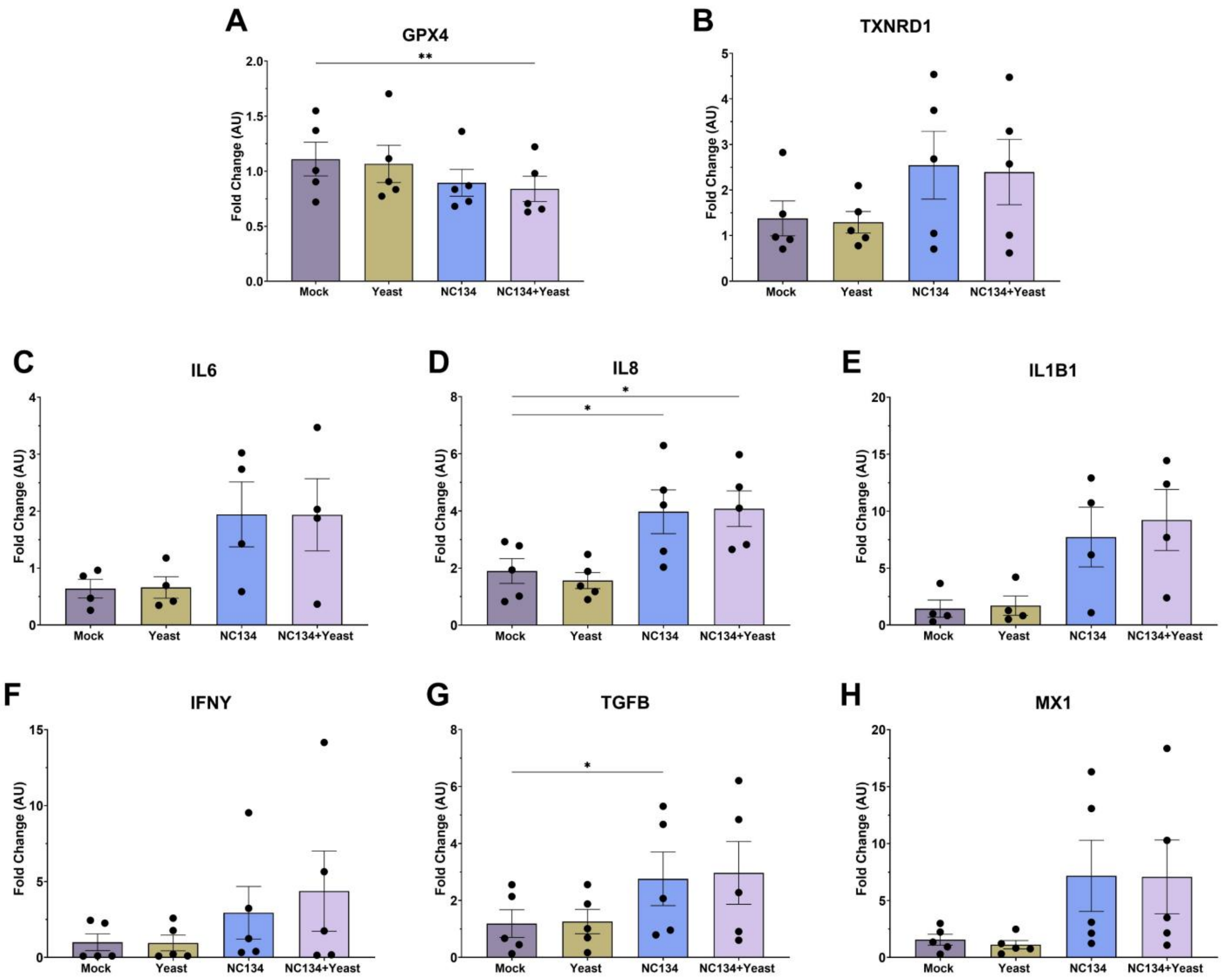

Figure S2. Gene expression of immune responses in PRRSV-2 NC134 and yeast-Se compound treated PAM, using NanoString technology. PAM were infected with NC134 MOI 0.5, treated with yeast-Se at 0.03 ppm, or left untreated (mock). Obtained values were normalized to the mean of mock values and shown as Fold Change (AU). Fold changes of (A) GPX4, (B) TXNRD1, (C) IL6, (D) IL8, (E) IL1B1, (F) IFNY, (G) TGFB, (H) MX1. All experiments were performed using between 4-5 biological replicates. Each symbol represents one biological replicate. Statistical significance was determined by comparing the means of all independent groups using One Way ANOVA. Results displayed showing mean and standard error of the mean (SEM). *p≤0.05, **p≤0.01.

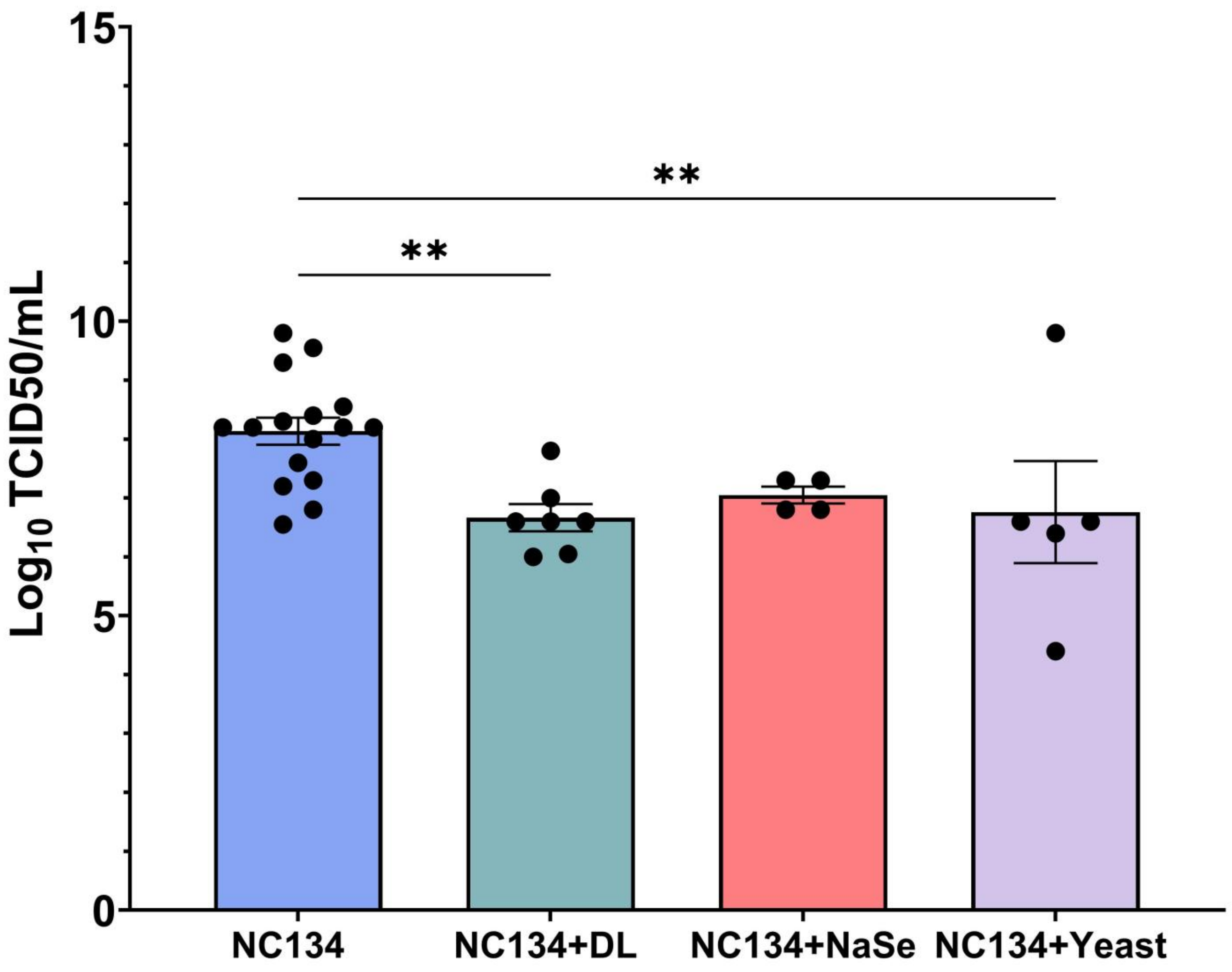

Figure S3. Measurement of viral titer of PRRSV-2 NC134 and Se-compound treated MA-104 cell line. Briefly, cells were infected with NC134 at MOI 0.5 and treated with organic or inorganic Se at 0.3 ppm concentration for 24 hrs. Supernatant collected for $TCID_{50}$ analysis. Experiments performed using between 4-7 technical replicates, mock=13. Each symbol represents one biological replicate. Statistical significance was determined by comparing the means of all independent groups using One Way ANOVA. Results displayed showing mean and standard error of the mean (SEM). $^{**}p \leq 0.01$.
