# Supplementary material for: Differential Effects of Selenium Compounds on Mitochondrial Function in PRRSV-Infected Porcine Alveolar Macrophages
Source: Viruses. 2025 Sep 26;17(10):1303. doi: 10.3390/v17101303 (PMC12568000; doi:10.3390/v17101303)
Supplement: Supplementary file 1 [file viruses-17-01303-s001.zip › Supplementary Table S1.pdf]

Table S1. NanoString custom codeset of 46 pig genes.

Gene	Accession No.	Class Name
C3	NM_214009.1	Endogenous
C5	NM_001001646.1	Endogenous
CASP3	XM_005671704.2	Endogenous
CD83	XM_001928655.4	Endogenous
CD86	NM_214222.1	Endogenous
CR1	XM_013979663.1	Endogenous
FCER1G	NM_001001265.1	Endogenous
FCGR1A	NM_001033011.1	Endogenous
FCGR2B	NM_001033013.2	Endogenous
FOS	NM_001123113.1	Endogenous
GPX4	NM_214407.1	Endogenous
HMGB1	NM_001004034.1	Endogenous
IFNA	NM_214393.1	Endogenous
IFNG	NM_213948.1	Endogenous
IFNGR1	NM_001177907.1	Endogenous
IFNb	NM_001003923.1	Endogenous
IKBKB	NM_001099935.1	Endogenous
IL 10	NM_214041.1	Endogenous
IL 12p35	NM_214013.1	Endogenous
IL 12p40/IL 23	NM_213993.1	Endogenous
IL 17	NM_001005729.1	Endogenous
IL 1A	NM_214029.1	Endogenous
IL 1B1	NM_214055.1	Endogenous
IL 6	NM_214399.1	Endogenous
IL 8	NM_213867.1	Endogenous
IRF1	NM_001097413.1	Endogenous
JAK2	NM_214113.1	Endogenous
JAK3	XM_003123500.3	Endogenous
MBL1	NM_001007194.3	Endogenous
MX1	NM_214061.1	Endogenous
NLRP3	NM_001256770.1	Endogenous
SERPING 1	NM_001123194.1	Endogenous
SOCS3	NM_001123196.1	Endogenous
TGFb	NM_214015.1	Endogenous
TNF	NM_214022.1	Endogenous
TREM1	NM_213756.1	Endogenous
TXNRD1	NM_214154.3	Endogenous
GAPDH	NM_001206359.1	Housekeeping
HMBS	NM_001097412.1	Housekeeping
HPRT1	NM_001032376.2	Housekeeping

RPL32	NM_001001636.1	Housekeeping
RPL4	XM_005659862.2	Housekeeping
RSP24	XM_001929385.6	Housekeeping
SDHA	XM_013992340.1	Housekeeping
TBP	XM_013991786.1	Housekeeping
TOP2B	NM_001258386.1	Housekeeping
